# The effect of spherical projection on spin tests for brain maps

**DOI:** 10.1162/IMAG.a.118

**Published:** 2025-08-21

**Authors:** Vincent Bazinet, Zhen-Qi Liu, Bratislav Misic

**Affiliations:** McConnell Brain Imaging Centre, Montréal Neurological Institute, McGill University, Montréal, Canada

**Keywords:** null models, spin test, spatial autocorrelation, significance testing, spherical projection

## Abstract

Statistical comparison between brain maps is a standard procedure in neuroimaging. Numerous inferential methods have been developed to account for the effect of spatial autocorrelation when evaluating map-to-map similarity. A popular method to generate surrogate maps with preserved spatial autocorrelation is the spin test. Here we show that a key component of the procedure—projecting brain maps to a spherical surface—distorts distance relationships between vertices. These distortions result in surrogate maps that imperfectly preserve spatial autocorrelation, yielding inflated false positive rates. We then confirm that targeted removal of individual spins with high distortion reduces false positive rates. Collectively, this work highlights the importance of accurately representing and manipulating cortical geometry when generating surrogate maps for use in map-to-map comparisons.

## 1 Introduction

Modern neuroimaging often necessitates evaluating the similarity between brain maps. Technological and data sharing advances have resulted in the proliferation of whole-brain maps of multiple structural and functional features (Markello et al., 2022), including gene expression (Akbarian et al., 2015; Hawrylycz et al., 2012; Markello et al., 2021), neurotransmitter receptors (Beliveau et al., 2017; Hansen et al., 2022; Nørgaard et al., 2021; Zilles & Palomero-Gallagher, 2017), synapse types (Zhu et al., 2018), cell types (Seidlitz et al., 2020; Vidal-Pineiro et al., 2020) and morphology (Scholtens et al., 2014), laminar differentiation (Paquola et al., 2019; Wagstyl et al., 2020), myelination (Burt et al., 2018; Glasser & Van Essen, 2011; Huntenburg et al., 2017; Whitaker et al., 2016), metabolism (Castrillon et al., 2023; Vaishnavi et al., 2010), and intrinsic dynamics (Frauscher et al., 2018; Gao et al., 2020; Murray et al., 2014; Shafiei et al., 2020, 2023; Wang, 2020). Against this backdrop, scientific discovery and inference typically involves a simple yet fundamental step: computing spatial correlations between pairs of brain maps. These comparisons are used to address two broad classes of questions. The first is to contextualize brain maps; for instance, asking whether an experimentally generated map (e.g., a cortical thickness contrast between patients and controls) is enriched for a particular micro-architectural feature (e.g. neurotransmitter receptors) (Hansen et al., 2022). The second is to identify ontological links between levels of description in the brain; for instance, asking whether a micro-scale feature (e.g., intracortical myelin) is related to a macro-scale feature (e.g. functional hierarchy) (Huntenburg et al., 2017).

Importantly, brain maps are spatially auto-correlated: areas that are spatially close to one another tend to be more similar than areas that are spatially distant from one another. A consequence of this is that observations are not independent from one another, violating the assumptions of both conventional parametric or non-parametric tests of significance (Alexander-Bloch et al., 2018; Burt et al., 2020). Specifically, spatial autocorrelation inherent in brain maps has been shown to yield spurious correlations and inflated p-values, leading to increased false positive rates (Koussis et al., 2025; Markello & Misic, 2021). In other words, the background influence of spatial autocorrelation in brain maps can lead to a spurious interpretation that two brain features are related to each other (Váša & Mišić, 2022). As a result, multiple methods have been developed to generate surrogate brain maps that attempt to preserve spatial autocorrelation in empirical maps and build null distributions for map-to-map correlation coefficients (Breakspear et al., 2004; Burt et al., 2018, 2020) (for a review, see Váša & Mišić (2022)).

Perhaps the most widely used method to generate surrogate maps with preserved spatial autocorrelation is a spatial permutation procedure that involves rotating spherical projections of brain maps (Alexander-Bloch et al., 2013, 2018). Specifically, feature values represented on a brain surface are projected to a sphere, subjected to random angular rotation, and then projected back onto the brain surface. Theoretically, this procedure should yield a new brain map in which the original feature values and their spatial autocorrelation are preserved, but their relationship with the underlying brain areas has been randomized. Since its inception, this intuitive and simple procedure—colloquially referred to as the “spin test”—has been readily adopted by the neuroimaging community, leading to numerous variants and implementations (Alexander-Bloch et al., 2018; Baum et al., 2020; Cornblath et al., 2020; Markello & Misic, 2021; Váša et al., 2018; Vázquez-Rodríguez et al., 2019).

Despite its popularity and conceptual appeal, multiple benchmarking studies have shown that in controlled simulated settings, the spin test does not perfectly control for false positives (Koussis et al., 2025; Markello & Misic, 2021). Here, we explore why this is the case. We first illustrate how the projection of brain surfaces to the sphere leads to deviations from the empirical spatial autocorrelation. We also show that these errors directly result in greater false positive rates. Finally, we show how targeted removal of high-error surrogate maps can be used to reduce the false positive rates.

## 2 Methods

### 2.1 Spatially autocorrelated maps

To evaluate the statistical performance of the spin test, we ran simulation experiments using spatially auto-correlated maps generated with the GSTools python toolbox (Müller et al., 2022). The spatial properties of these maps were derived from a Gaussian variogram model defined as follows:

$$\gamma (h,l)=\sigma^{2}[1-e^{-{\left(\frac{\left|h\right|}{l}\right)}^{2}}],$$ (1)

where h denotes the Euclidean distance between vertices. The parameter s is a rescaling factor, set to the toolbox’s default value of $\frac{\sqrt{\pi }}{2}$,  l denotes the length of the model, and $\sigma^{2}$ denotes the variance of the model. From this variogram model, a random map u(x) , spatially autocorrelated in Euclidean space, can be generated using the following calculation, known as the randomization method (Heße et al., 2014; Kraichnan, 1970):

$$u(\text{x})=\sqrt{\frac{\sigma^{2}}{N}}\sum_{i=1}^{N}\left(Z_{1,i}\cdot cos({\text{k}}_{\text{i}}\text{x})+Z_{2,i}\cdot sin({\text{k}}_{\text{i}}\text{x})\right),$$ (2)

where N is the number of Fourier modes used. Here, N was set to 1 000. $Z_{i,j}$ are random samples drawn from a normal distribution and ${\text{k}}_{\text{i}}$ are samples from the spectral density distribution of the variogram model. The range of the variogram (i.e., distance at which the maximal variance is attained) approximately corresponds to $\sqrt{3}\frac{l}{s}$. Thus, the larger the length parameter, the greater the spatial autocorrelation of the maps generated. To evaluate the performance of the spin test across levels of autocorrelation, 1 000 random fields were generated for 6 length values ranging from 1 to 50. The same $Z_{i,j}$ values were used across length to allow consistent comparisons across autocorrelation levels. Supplementary Figure S6 shows the variograms of the model for each length parameter. To compare the performance of the spin test across surfaces, maps were generated on two different meshes of the left hemisphere’s fsaverage5 template (Fischl, Sereno, Tootell, et al., 1999), namely the pial and the spherical surface meshes.

### 2.2 Moran’s I

The spatial autocorrelation of the maps considered in this work was quantified using Moran’s I (Cliff & Ord, 1981; Moran, 1950). A Moran’s I of 0 indicates that there is no spatial autocorrelation in the data, a Moran’s I greater than 0 indicates that there is spatial autocorrelation in the data (values of proximal vertices are more similar than values of distant vertices) and a Moran’s I less than 0 indicates the presence of anti-correlation (values of distant vertices are more similar than values of proximal vertices). The Moran’s I is defined as follows:

$$I=\frac{1}{W_{0}}\sum_{ij}w_{ij}z_{i}z_{j},$$ (3)

where $w_{ij}$ corresponds to the spatial weight quantifying the spatial relationship between vertices i and j, $W_{0}$ corresponds to the sum of all weights $w_{ij},$ and $z_{i}$ correspond to the standardized value of a brain map u at vertex i:

$$z_{i}=\frac{u_{i}-\bar{u}}{\sigma }.$$ (4)

The spatial weight for a pair of vertices i and j was defined as the inverse of the Euclidean distance between them. The resulting weight matrix was then row-normalized.

### 2.3 Local distortions in spatial autocorrelation

The local contribution of a map’s values to the Moran’s I statistic can be evaluated with the local Moran’s I (Anselin, 1995), defined for a given vertex i as:

$$I_{i}=z_{i}\sum_{j}z_{j}w_{ij},$$ (5)

where $z_{i}$ and $z_{j}$ are standardized values of a map u. The relationship between the local I statistic and the Moran’s I is given by:

$$I=\frac{1}{W_{0}}\sum_{i}I_{i}.$$ (6)

When the weight matrix is row-normalized, the Moran’s I corresponds to the mean of the local Moran’s I statistics across vertices. To understand the effects of local distortions on spatial autocorrelation, we can evaluate the difference in local Moran’s I values between the original and rotated maps, which can be defined as:

$$\Delta \;I_{i}=z_{i}\sum_{j}z_{j}\left(w_{ij}^{P}-w_{ij}^{P^{\prime }}\right).$$ (7)

${\text{W}}^{P}$ and ${\text{W}}^{P^{\prime }}$ correspond to the weight matrices associated with the original and rotated pial surfaces, respectively.

The local effects of distortions on spatial autocorrelation can be visualized with a modified version of the Moran scatterplot. A Moran scatterplot shows the relationship between standardized values at each vertex (x-axis) and the weighted average of the standardized values in the neighborhood of each vertex (y-axis), with the product of the two axes giving the local Moran’s I of each vertex (Anselin, 1995). The modified Moran scatterplot still shows the standardized values of a map u on the x-axis and the weighted average of the standardized values of u on the x-axis, but the weights are now defined as the local distortions in the weight matrices ${\text{W}}^{P}$ and ${\text{W}}^{P^{\prime }}$ . From the equation above, we find that the product of the two axes gives us the difference in local Moran’s I at each vertex.

### 2.4 The spin procedure

The results presented in this work relied on the fsaverage5 cortical mesh from FreeSurfer (Fischl, 2012). Spatially autocorrelated maps generated on the pial surface of the brain were first projected to the spherical mesh. This step was not necessary for spatially autocorrelated maps generated directly on this spherical mesh. Then, random rotations were applied to the spherical mesh using QR decomposition. The vertices on the rotated mesh were then matched to vertices on the original spherical mesh using a nearest neighbor approach. This process was repeated until a total of 10 000 realization (spins) were generated. For all experiments, only a subset of 1 000 spins were used. The 10 000-null ensemble was initially sampled at random. Then, for the later experiments, a certain percentage, ranging from 0 to 90%, of the lowest quality spins were removed from this null ensemble, and 1 000 spins were then sampled from this thresholded ensemble.

### 2.5 Statistical performance of the spin test

The statistical performance of the spin test was evaluated using the spatially autocorrelated maps described in section 2.1. For each spatial autocorrelation level (defined by the length parameter), a total of 1 000 maps were generated. The Pearson correlation between each pair of maps {X,  Y} was computed, and its significance was evaluated using the spin test. Namely, a total of 1 000 random rotations were applied to the map X. Each rotated map was then correlated with the map Y to generate a null distribution of correlation coefficients. The p-value of the correlation coefficient between {X,  Y} was then evaluated relative to this null distribution. For each map X, this procedure was repeated across all maps Y, resulting in a distribution of 999 p-values. The false positive rate of X was then computed as the ratio of significant relationships (p<0.05 threshold).

### 2.6 Quantifying the quality of spins

The spin procedure involves projecting a cortical surface (e.g., pial) P to a sphere S, rotating the sphere S to a sphere $S^{\prime }$ , then projecting the sphere $S^{\prime }$ back to a cortical surface $P^{\prime }$ . By concatenating all transformations, we get a mapping from a cortical surface P to a cortical surface $P^{\prime }$ . The quality of this mapping between the two cortical surfaces was quantified as the Pearson correlation between the original and transformed distance matrices (Supplementary Fig. S3).

### 2.7 Empirical brain maps

To better understand the practical implications of our findings, we evaluated the Moran’s I of empirical brain maps from the *neuromaps* toolbox (Markello et al., 2022). We included maps that were available in a surface-based coordinate system. In total, we included 28 brain maps from 6 different biological categories including gene transcription (gene PC1 (Hawrylycz et al., 2012)), neurotransmitter receptors and transporters (5HT1a, 5HT1b, 5HT2a, 5HT4, 5HTT (Beliveau et al., 2017), and GABAa (Nørgaard et al., 2021)), MEG (alpha, beta, delta, low gamma, high gamma, theta power distribution (Shafiei et al., 2022; Van Essen et al., 2013) and intrinsic timescale Shafiei et al. (2023)), structural (myelin and cortical thickness (Glasser et al., 2016)), functional (homology (Xu et al., 2020), gradient (Margulies et al., 2016), intersubject variability (Mueller et al., 2013) and sensory-association axis (Sydnor et al., 2021)), metabolism (cerebral blood volume, cerebral blood flow, metabolic rate of oxygen, metabolic rate of glucose consumption (Vaishnavi et al., 2010)) and expansion (evolutionary cortical expansion (Xu et al., 2020), cortical areal scaling during development for the HCP, NIH, and PNC datasets (Reardon et al., 2018)). Each brain map was resampled to fsaverage5 with multimodal surface matching (Robinson et al., 2014). The spatial weight used to compute the Moran’s I of brain maps was defined, for each pair of vertex i and j, as the inverse of the geodesic distance between them. The resulting matrix was then row-normalized.

## 3 Results

### 3.1 Spherical projections inflate false positive rates

**Fig. 1.**
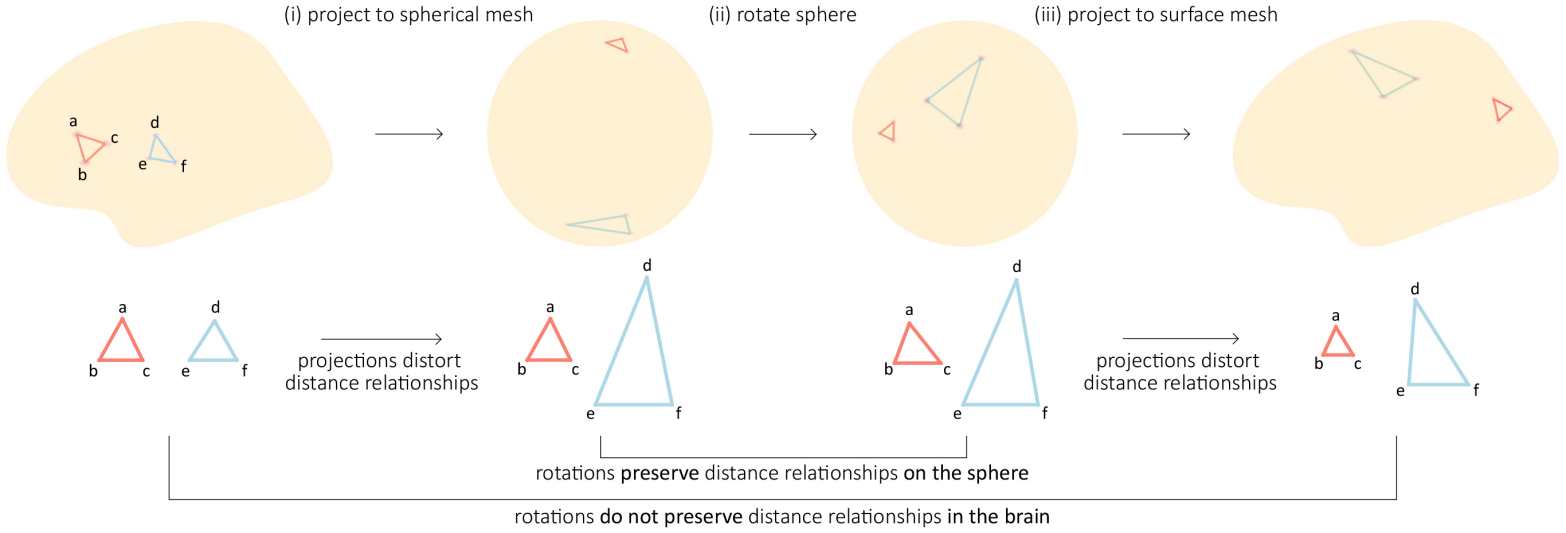
Spherical projections distort distance relationships. The spin procedure consists of three main steps: (i) projecting a brain map to a spherical mesh, (ii) rotating the sphere, and (iii) projecting the brain map back to the brain surface. The projection to a spherical mesh distorts distance relationships between vertices of the cortical surface mesh. For instance, the vertices a, b, and c (red triangle) as well as d, e, and f (blue triangle) are equally distant from one another on the cortical mesh but following the transformation, some distance relationships are not preserved. Namely, vertices d, e, and f are significantly more distant from one another, with vertex d farther away from vertices e and f. The rotation of the sphere, being an isometry, preserves distance relationships on the sphere. The projection from the sphere back to the brain further distorts distance relationships. For instance, vertices a, b, and c are closer to one another, while vertex d is now closer to vertex e than vertex f. Overall, the spin procedure does not preserve distance relationships in the brain.

The main goal behind rotating the brain is to generate surrogate brain maps that preserve the spatial structure of the original data. We start out by developing intuition about why projecting surface maps to a sphere distorts distance relationships between vertices. Figure 1 (left) shows two sets of equally distant vertices on a brain surface, shown in red and blue. Projecting a brain surface causes distortions (Fischl, Sereno, & Dale, 1999), approximately preserving some distance relationships (red) but disrupting others (blue). A rotation on the sphere is an isometry, meaning that it is a distance-preserving transformation. As a result, rotations of the spherical representation perfectly preserve distance relationships among the vertices (Fig. 1, middle). Projecting the spherical representation back to a brain surface then erroneously preserves distortions and introduces additional ones (Fig. 1, right). In other words, the entire procedure does not necessarily preserve distance relationships in the brain.

**Fig. 2.**
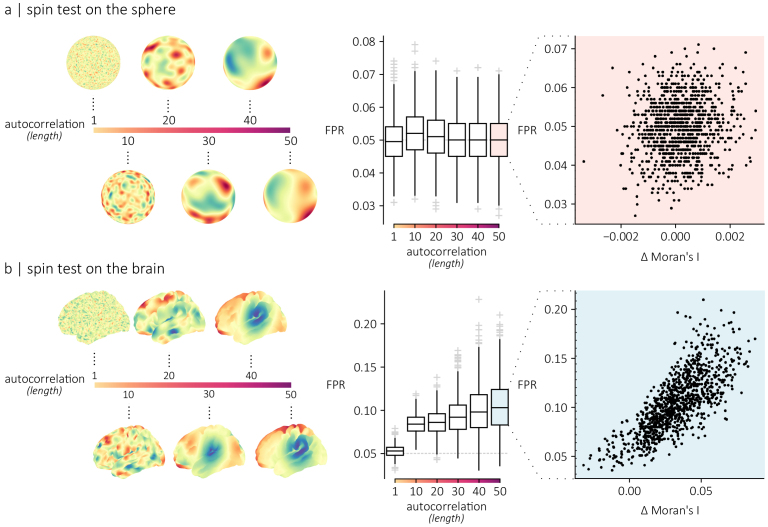
Spherical projections inflate false positive rates | The statistical performance of the spin test was evaluated using spatially autocorrelated maps generated by a Gaussian covariance model with a length parameter defining the spatial extent of the process. A sample of 1 000 maps was generated for six different levels of spatial autocorrelation (lengths) on either a spherical surface (a) or on a cortical surface (b). For each level of spatial autocorrelation, individual maps were correlated with the remaining 999 maps. The significance of each correlation coefficient was evaluated using the spin test. The false positive rate (FPR) was then quantified for each map individually. The boxplots show the distribution of false positive rate for each map, across lengths. The scatterplot shows the relationship between the false positive rate of a map and its average deviation in Moran’s I relative to its rotated versions (empirical−rotated ).

Figure 2 illustrates the effects of these distortions on the spin test. To quantify the statistical performance of the spin test, we run ground-truth simulations in which we compute false positive rates (FPRs) when correlating pairs of spatially autocorrelated random maps (Burt et al., 2020; Markello & Misic, 2021) (see section 2). Briefly, we used a spatial random field model to generate random maps that are spatially autocorrelated on the sphere (Fig. 2a, left) and random maps that are spatially autocorrelated on the brain’s surface (Fig. 2b, left). By tuning the length parameter of the model, we control the spatial autocorrelation of the generated maps. We generate 1 000 maps with a length parameter ranging from 1 mm to 50 mm. Then, for each random map, we compute the Pearson correlation between itself and the remaining 999 maps, and evaluate the significance of this Pearson correlation with a spin test, obtaining a total of 999 p-values for each random map. Setting the significance threshold to 0.05, we quantify the false positive rate of the statistical test for each random map as the proportion of correlations with a *p*-value below the significance threshold. Figure 2a (middle) shows that, on average, the false positive rate for random maps generated on a sphere is 0.05 regardless of the spatial autocorrelation, confirming that the randomization (and thus, the significance test) works as expected. Figure 2b (middle) shows that for maps that are spatially autocorrelated on the brain’s surface, the false positive rates increase as the spatial autocorrelation increases.

We then used the Moran’s I statistic, a measure of spatial autocorrelation (Cliff & Ord, 1981; Moran, 1950), to quantify the mean difference in spatial autocorrelation between each random map and their rotated versions. We show that the distortions resulting from the spin procedure lead to discrepancies in spatial autocorrelation between the original and rotated maps—see Supplementary Figure S1 for a detailed explanation—and we relate these differences to the statistical performance of the spin test. For maps generated on the spherical mesh, we find that the Moran’s I of the original map is well preserved on average, with a mean absolute difference of 0.0007 (Fig. 2a, right). Consistent with the intuition developed in Figure 1, we find that the Moran’s I of the maps generated on the cortical mesh is not perfectly preserved after the rotations, with a mean absolute difference of 0.03 (Fig. 2b, right). Importantly, we also find that the false positive rates are proportional to the average deviation in spatial autocorrelation between the original and rotated maps (r=0.76 ). In other words, the statistical bias in the spin test depends on the deviation in spatial autocorrelation relative to rotated surrogates. To better understand the practical implications of this finding, we evaluated the Moran’s I of empirical brain maps from the *neuromaps* toolbox (Markello et al., 2022), and calculated their standardized value relative to the Moran’s I of rotated surrogates of each map (Supplementary Fig. S2). Standardized Moran’s I values vary extensively across empirical maps, with the 5HTT density and intersubject functional connectivity variability maps showing the largest standardized I values ($z_{I}=2.10$ and $z_{I}=2.17$ respectively), and the cerebral blood volume map showing the smallest standardized I value ($z_{I}=-1.63$ ). In summary, projecting brain maps to a spherical surface distorts distance relationships, introducing statistical bias in the spin test.

### 3.2 Targeted removal of poor nulls improves performance

If a specific spherical rotation (realization of the spin test null model) does not adequately preserve the spatial relationships in brain maps, then a straightforward solution would be to remove it from the population of nulls. To quantify how well a rotation preserves distance relationships among vertices, we compute the correlation between vertex-to-vertex distance matrices of original and rotated surfaces (Supplementary Fig. S3). Supplementary Figure S4 confirms that maps permuted with rotations that do not preserve distance relationships are those whose spatial autocorrelation deviates the most from the original map. We then repeat the experiments from the previous section, but gradually remove surrogate maps that have been permuted with poorly aligned spherical rotations.

**Fig. 3.**
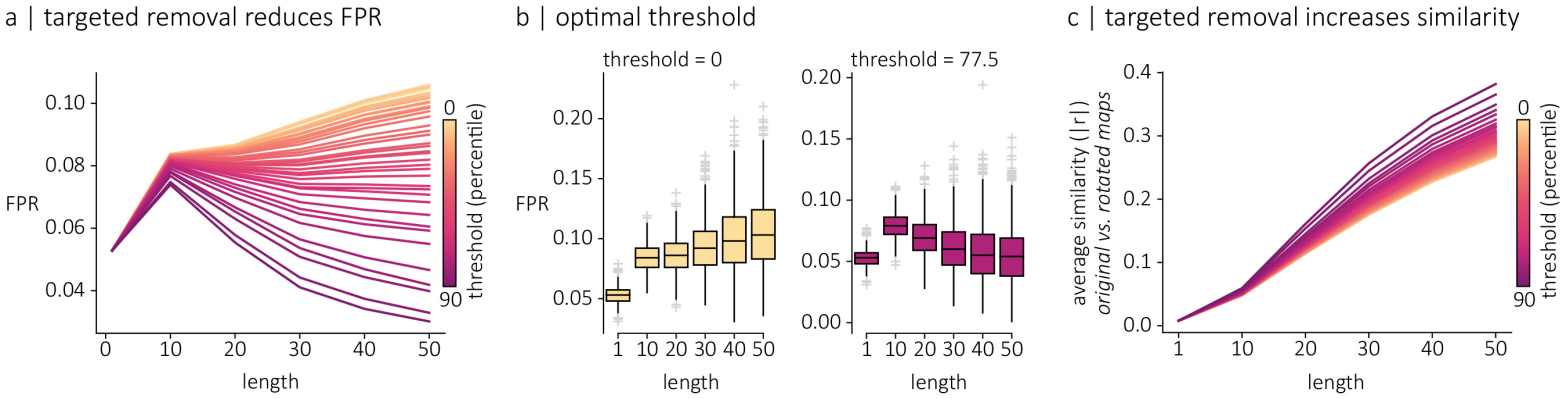
Targeted removal of poor nulls improves performance. (a) Statistical performance of the spin test (FPR) following the targeted removal of suboptimal rotations. Individual lines represent the average FPR across simulated maps, for each autocorrelation level. Line colors indicate the threshold (in percentile) for the removal of rotations, ranging from 0 to 90. (b) Distribution of false positive rates quantified for each map individually, across autocorrelation levels when no threshold is applied (left) and when a threshold of 77.5 is applied (right). (c) Average similarity (absolute Pearson coefficient) between original and permuted maps for each autocorrelation levels, with each line representing a different threshold for the removal of rotations.

Figure 3a shows FPRs when applying this removal heuristic. As before, we quantify FPRs at varying levels of spatial autocorrelation. Different lines illustrate performance at different thresholds, from 0% removal to 90% removal. See Supplementary Figure S5 for the correlation values associated with each threshold. Note that, to ensure that all null populations are of equal size, we always sample 1  000 nulls out of the original 10,000-null ensemble (see section 2). As poorly aligned spins are gradually removed, we observe lower FPRs. In these specific simulations (though the result may not generalize to all situations), we observe 5% FPRs at approximately 77.5% removal (Fig. 3b), which corresponds to a threshold set to r=0.64 . At greater removal rates, FPRs dip below the desired 5% level; Figure 3c shows that this might be because the remaining null realizations resemble the original map too closely and do not sufficiently sample the null space. In summary, removing poorly aligned rotated maps reduces the risk of Type I (false positive) errors, potentially resulting in improved performance of spatial permutation tests but entails heuristic intervention in the sampling procedure that increases the risk of Type II (false negative) errors.

## 4 Discussion

Spatial correlations between brain maps are a fundamental component of scientific discovery in brain imaging. Here we explored why a popular spatial null model for assessing statistical significance does not adequately control false positive rates. We then show how a straightforward modification of the method can reduce false positive rates.

We find that false positive rates in spatial permutation tests can be traced back to the initial step of projecting a brain surface to a sphere. Inflating a brain surface to a sphere induces metric distortions due to cortical curvature (Fischl, Sereno, & Dale, 1999). As a result, patterns of autocorrelation on a brain surface are distorted when projected to a sphere. Importantly, we confirm that subsequent angular rotations of the spherical representation are not problematic, being isometric transformations. In this work, we rely on the fsaverage5 template from Freesurfer (Fischl, 2012). This template was constructed by averaging the aligned spherical meshes of 40 subjects (Fischl, Sereno, Tootell, et al., 1999), which were mapped to the sphere via the minimization of a distance-preserving (i.e. metric) energy functional. Our results may differ from those obtained using alternative spherical mapping algorithms that for instance rely on area-preserving (authalic) mappings (Zou et al., 2011), angle-preserving (conformal) mappings (Gu et al., 2004), or that incorporate additional isometry correction processes (Yotter et al., 2011). Importantly, an isometric mapping is theoretically impossible given the different Gaussian curvature of the cortical and spherical surfaces (Gauss, 1828). Thus, spherical mappings inevitably alter the spatial relationship between vertices.

This work is part of an emerging appreciation of the fact that the brain surface is irregular. Namely, the standard preprocessing step of mapping volumetric voxels to a highly folded surface yields uneven surface vertex spacing, such that neighboring vertices are physically closer in sulci than in gyri. This uneven sampling can be suboptimal (Feilong et al., 2024) and can introduce a “gyral bias” in fMRI analyses (Jeganathan et al., 2025). The distortions that we observe when mapping cortical surfaces to spheres and which lead to inflated *p*-values in the spin test are directly related to this uneven sampling. In this sense, our findings point to a need to develop methods that more accurately represent and manipulate cortical geometry. How to quantify features of local geometry on the brain surface, such as autocorrelation (Leech et al., 2024) or wave patterns (Pang et al., 2023), is an open question in the field.

We explore a simple heuristic to reduce the effect of ill-preserved spatial autocorrelation on false positive rates. We find that some realizations of spins are better than others, and trace the inflated false positive rates to specific realizations that poorly preserve spatial autocorrelation of the empirical map. We show that targeted removal of deviant individual null realizations can reduce false positive rates to desirable levels. Conceptually, this procedure can be thought of as performing post hoc quality control on the spatial null generating process. Ultimately, the goal of the spatial randomization procedure is to sample the wider null space of brain maps with similar autocorrelation (Váša & Mišić, 2022); the proposed “fix” helps to prune and narrow down this space such that it only includes realizations of sufficient quality, that is, sufficiently similar autocorrelation to the empirical map.

Going forward, what spatial autocorrelation-preserving method should researchers use? The null hypothesis of the spin test is that random rotations explain the alignment between a pair of brain maps and computing a *p*-value therefore only necessitate random sampling from the uniform distribution on the set of rotation matrices (Alexander-Bloch et al., 2018). As such, they are fast, easy to apply, and the same “spin” can be applied uniformly to all maps in a given sample. In contrast, they do not perfectly preserve spatial autocorrelation on cortical surfaces. We propose a solution for reducing false positive rates, but it requires additional experimenter intervention that can potentially bias sampling of the null space. This is an active field of research and there exist numerous alternative methods to randomize brain maps while preserving spatial autocorrelation (Burt et al., 2018, 2020; Koussis et al., 2025; Wagner & Dray, 2015). They each rely on different definitions of spatial autocorrelation and employ distinct optimization heuristics but none perfectly control for false positive rates (Koussis et al., 2025; Markello & Misic, 2021). Importantly, the aforementioned models assume that autocorrelation patterns are homogeneous across the cortex, yet this assumption is likely inaccurate (Hayasaka et al., 2004; Leech et al., 2023) and understanding how spatial heterogeneity affects statistical inferences will be essential for the development of sound statistical methods (Leech et al., 2024). Alternative methods for evaluating correspondence between modalities (Hu et al., 2022) and alternative statistical tests that do not rely on the generation of spatial autocorrelation-preserving surrogate maps should also be considered (Weinstein et al., 2021, 2022, 2024). Ultimately, the field is still in its infancy and it is too early to draw overly prescriptive conclusions. More research is warranted in this domain, not only on null models but on the geometric features of the brain more generally (Pang et al., 2023).

## Supplementary Material

Supplementary Material

## Data Availability

The data and code used to conduct the analyses and generate the figures presented in this paper are available at https://github.com/netneurolab/bazinet_spins and directly rely on the following open source Python packages: NumPy (Harris et al., 2020), Scipy (Virtanen et al., 2020), Matplotlib (Hunter, 2007), GSTools (Müller et al., 2022), NiBabel (Brett et al., 2024), PyVista (Sullivan & Kaszynski, 2019), and neuromaps (Markello et al., 2022).
