# Supplementary material for: The effect of spherical projection on spin tests for brain maps
Source: Imaging Neurosci (Camb). 2025 Aug 21;3:IMAG.a.118. doi: 10.1162/IMAG.a.118 (PMC12371478; doi:10.1162/IMAG.a.118)
Supplement: Supplementary Material [file IMAG.a.118_supp.pdf]

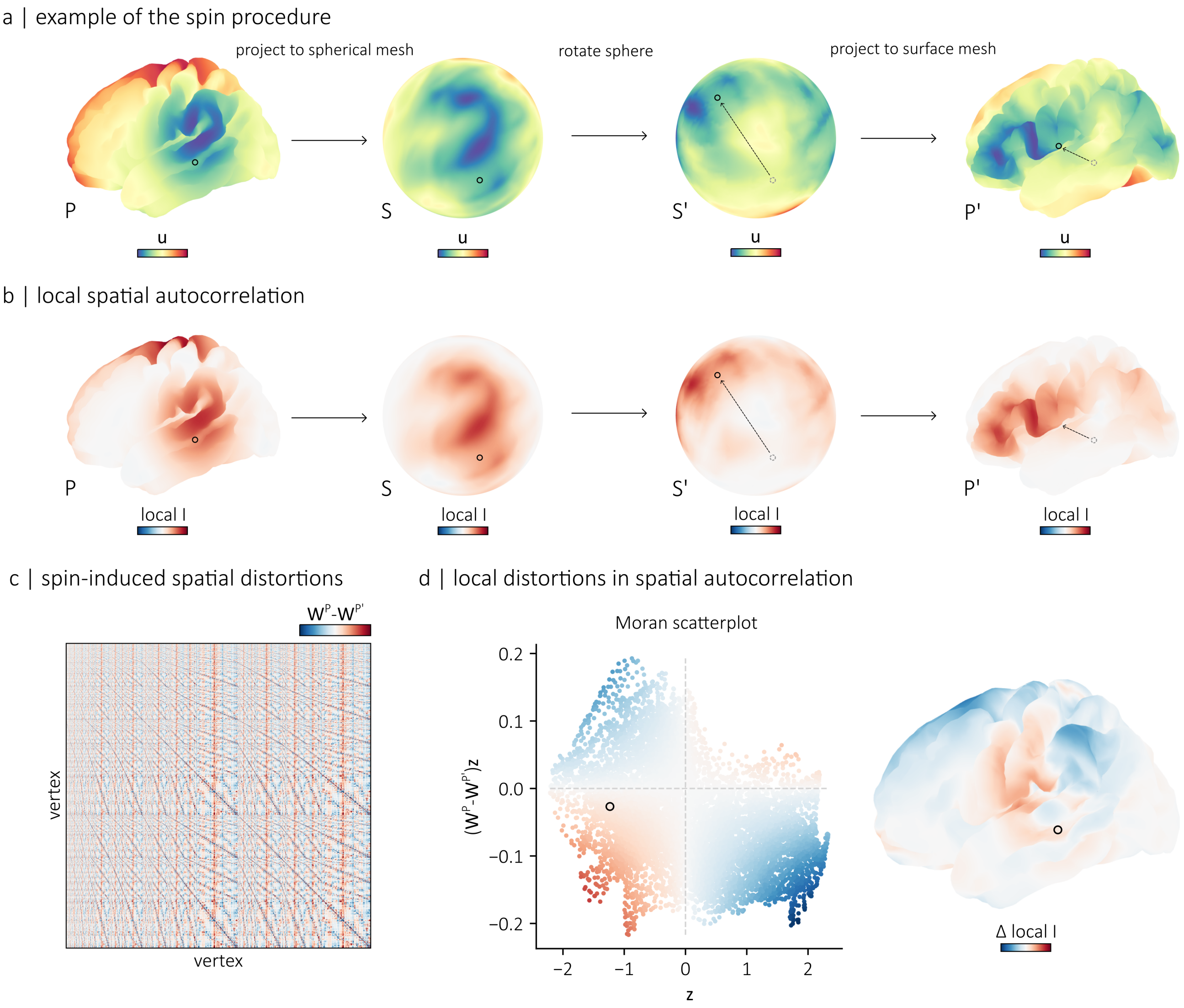

Figure S1 **Impact of distortions on local spatial autocorrelation** | We provide an example of the spin procedure for a random map **u** generated on the cortical surface using a Gaussian variogram model (length=50) and describe its effect on local spatial autocorrelation. (a) The spin procedure consists of three main steps: (i) projecting the brain map to a spherical mesh, (ii) rotating the sphere and (iii) projecting the brain map back to the brain surface. (b) The contributions of individual vertices to the Moran's I metric can be evaluated using the local Moran's I. We show these contributions for each step of the spin procedure. (c) Ultimately, the spin procedure results in spatial distortions, which can be visualized by comparing the original weight matrix (inverse of distance) to the permuted weight matrix ($\mathbf{W}^P$ - $\mathbf{W}^{P'}$). (d) The local variations in Moran's I following the spin procedure (Δ local I) can be visualized in a modified Moran scatterplot looking at the relationship between the standardized value at a vertex (**z**; x-axis) and the weighted average of the standardized values, with weights corresponding to the local distortions in the neighborhood of that vertex (y-axis). See *Methods* for more details.

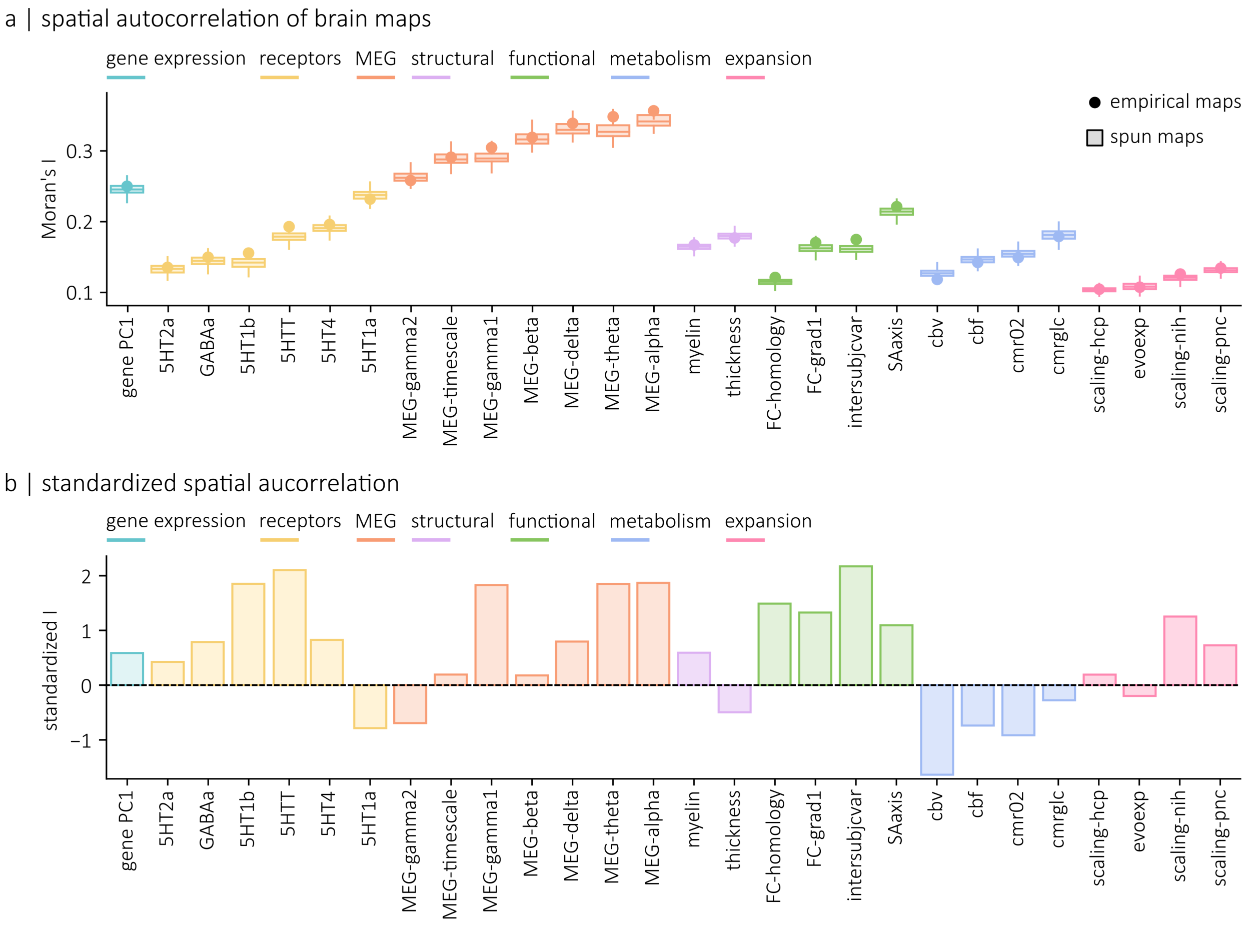

Figure S2 **Impact of distortions on spatial autocorrelation of empirical brain maps** | (a) We evaluated the spatial autocorrelation of empirical brain maps from the neuromaps toolbox using the Moran's I statistic. The Moran's I of the empirical brain maps (points) are compared to the Moran's I of rotated surrogates (boxplots). (b) For each brain map, we evaluated their standardized Moran's I relative to the distribution of Moran's I from rotated surrogate maps.

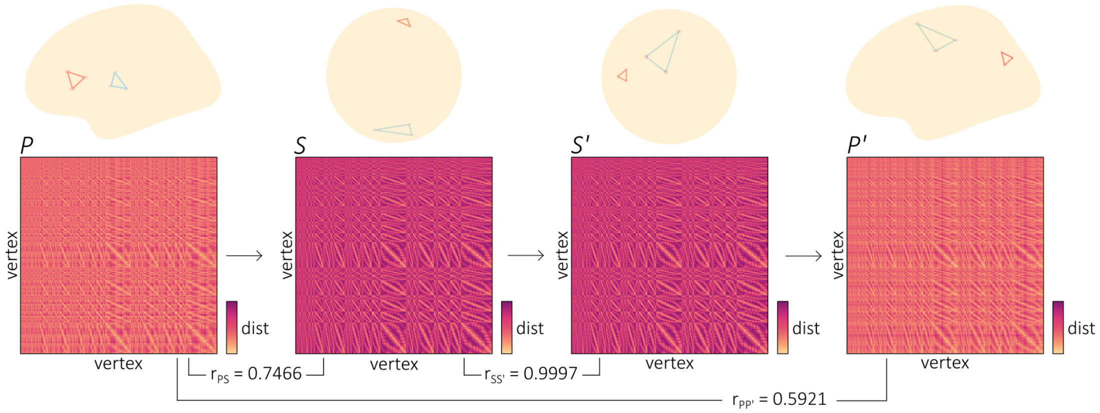

Figure S3 **Quality of spins** | The spin procedure involves projecting a cortical surface P to a sphere S, rotating the sphere S to a sphere S', then projecting the sphere S' back to a cortical surface P'. The quality of each transformation can be quantified as the Pearson correlation between the distance matrix in the original space and the distance matrix in the transformed space. For the first transformation $P \rightarrow S$, we have $r_{PS} = 0.7466$. The fact that r_{PS} is not equal to 1 indicates that the projection is not isometric and that distortions have been introduced. For the second transformation $S \rightarrow S'$, we evaluated the quality of a random rotation obtained via QR decomposition and found: $r_{SS'} = 0.9997$, which confirms that a rotation of the sphere is an isometry. Note that $r_{SS'}$ is not exactly equal to 1 because the spherical mesh is a recursively subdivided icosahedron, and not a perfect sphere. By concatenating each transformation, we ultimately obtain a transformation $P \rightarrow P'$, which captures the entire spin procedure. For that specific spin instance, we have $r_{PP'} = 0.5921$.

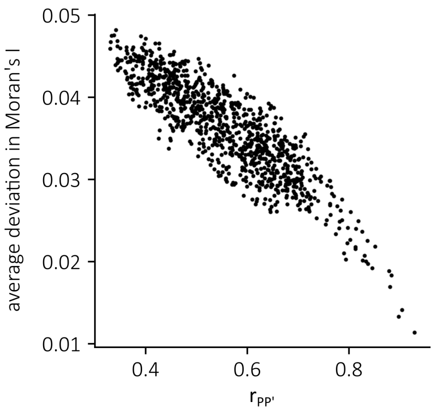

Figure S4 **Rotations of poorer quality are associated with larger deviations in Moran's I** | The quality of each rotation was evaluated as the Pearson correlation between the distance matrix in the original space and the distance matrix in the transformed space ($r_{PP'}$). The quality of each rotation was then compared to the average deviation in Moran's I between original maps and maps permuted with that specific rotation. Namely, each rotation was applied to 1 000 spatially autocorrelated maps (length=50) and, for each map, the absolute difference between its Moran's I following the rotation and its Moran's I prior to the rotation was evaluated. For each rotation, its average deviation in Moran's I was then computed as the mean of this absolute difference, across all maps. We find a strong negative relationship between the quality of a rotation and the average deviation in Moran's I associated with that rotation ($r = -0.88$).

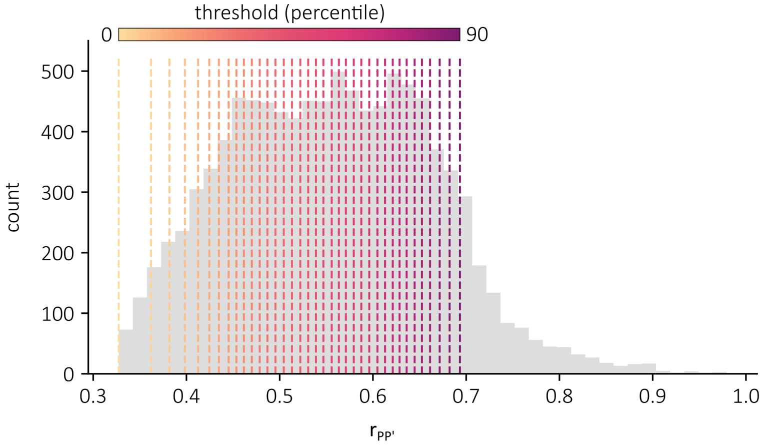

Figure S5 **Distribution of rotation quality correlations** | The quality of each rotation was evaluated as the Pearson correlation between the distance matrix in the original space and the distance matrix in the rotated space ($r_{PP'}$). We show the distribution of correlation values obtained for all rotations (N=10 000). The vertical lines identify the correlation coefficients associated with each threshold used for the targeted removal of poorly aligned spins (from 0th percentile to 90th percentile).

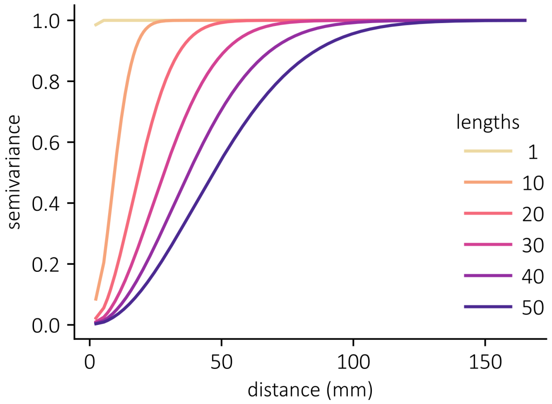

Figure S6 **Gaussian variogram models** | Each colored line represents the Gaussian variogram model for a specific length parameter value, ranging from 1 (yellow) to 50 (purple). The y-axis outlines the semivariance – half of the average squared difference between values separated by a distance h [1], itself plotted on the x-axis.

[1] Matheron, G. (1963). Principles of geostatistics. *Economic geology*, 58(8):1246–1266.
